# Retrospective Analysis of Persistent Clonal *Salmonella enterica* Strains of Various Serovars in Commercial Swiss Broiler Farms

**DOI:** 10.1002/mbo3.70149

**Published:** 2025-11-24

**Authors:** Maher Alsaaod, Marc J. A. Stevens, Nicole Cernela, Jule Anna Horlbog, Roger Stephan, Sarah Albini

**Affiliations:** ^1^ National Reference Center for Poultry and Rabbit Diseases (NRGK), Institute for Food Safety and Hygiene, Vetsuisse Faculty University of Zurich Zurich Switzerland; ^2^ Institute for Food Safety and Hygiene, Vetsuisse Faculty University of Zurich Zurich Switzerland; ^3^ National Reference Center for Enteropathogenic Bacteria and Listeria (NENT), Institute for Food Safety and Hygiene, Vetsuisse Faculty University of Zurich Zurich Switzerland

**Keywords:** broiler chicken, farm level, persistent clones, public health, *Salmonella* Idikan, *Salmonella* serovars, whole‐genome sequencing

## Abstract

Detection of *Salmonella* Enteritidis (SE) and *S*. Typhimurium (STm) in broiler holdings is regulated by European and Swiss law to ensure public health. Persistence of *Salmonella* in broiler houses may jeopardize this goal. The aim of this study was to analyze whether non‐SE/STm isolated from boot socks were of clonal origin. Four *Salmonella* serovars from 11 broiler houses from 10 Swiss farms were selected: *S*. Infantis, *S*. Livingston and *S*. Welikade (meat integration A) and *S. enterica* subsp. e*nterica* 13,23:i:‐ (integration B). The genetic relationship was evaluated by whole‐genome sequencing (WGS) and core genome multilocus sequence typing (cgMLST)‐based tree analysis, with a cluster being defined as < 8 cg alleles differences. The isolates of *S*. Infantis and *S*. Livingston, respectively, were shown to belong to the same serovar‐specific clusters (range: 1–7 cg alleles differences), suggesting that the *Salmonella* strains persisted in the respective broiler houses. *S*. Welikade, however, showed 8–11 cg alleles differences among isolates, indicating either a reintroduction of similar but not clonal strains into the houses due to insufficient biosecurity, or the evolution of a persistent strain. Remarkably, all isolates of *S*. 13,23:i:‐ from integration B from 2013 to 2024 were clonal, suggesting dispersal and persistence in the broiler integration. The clonality of analyzed strains suggests that *Salmonella* can persist on farms or integration level despite disinfection after each production cycle. Hence, improved farm and vehicle cleaning and disinfection practices are essential to ensure that the next flock is not exposed to non‐SE/STm *Salmonella* serovars.

## Graphical abstract

Identical clones of non‐Enteritidis/Typhimurium *Salmonella* serovars (non SE/STm), repeatedly isolated from the same broiler houses over months and years, suggesting their persistence in the farm environment, despite disinfection after each production cycle. This study stresses the utmost importance of on‐farm biosafety measures as fundamental elements to protect public health.

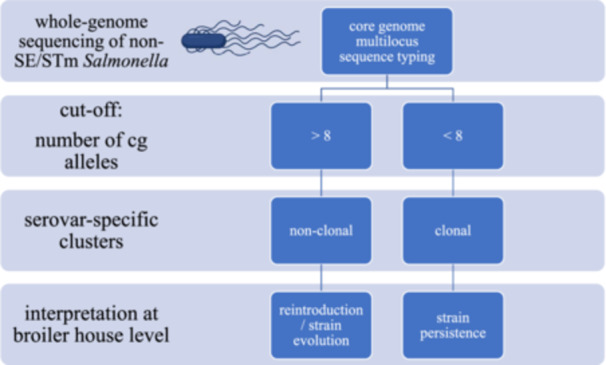

## 1 Introduction

Poultry meat is among the primary sources of *Salmonella* infection worldwide (Koutsoumanis et al. 2019; Cota et al. 2024; Logue et al. 2024), with the economic burden estimated to be more than $2.8 billion annually in the United States (Scharff 2020). In 2023, salmonellosis remained the second most reported zoonotic disease after campylobacteriosis in the European Union (EU) and Switzerland, with 77,486 and 1823, laboratory‐confirmed human cases, respectively (Anonymous 2024; The European Union One Health 2023 Zoonoses report 2023 Zoonoses report 2024). *Salmonella enterica* subspecies *enterica* serovar Enteritidis (*S*. Enteritidis, SE), *S*. Typhimurium (STm), and *S*. Typhimurium monophasic variant (mSTm) were the most commonly reported serovars responsible for human salmonellosis in the EU in 2021 (The European Union One Health 2021 Zoonoses Report 2021 Zoonoses Report 2022). In broilers, STm and SE are generally the most common and most extensively researched serovars (Nazari Moghadam et al. 2023).

Entry of *Salmonella* into a broiler farm can occur by horizontal transmission via environmental sources, such as contaminated feed or drinking water, by staff members and their clothes, stable equipment, transmission vehicles and by rodents (Liljebjelke et al. 2005; Meerburg and Kijlstra 2007; Dar et al. 2017; Brandenburg et al. 2024). It has further to be taken into account that *Salmonella* from preceding flocks might survive in the poultry house or its environment (Voss‐Rech et al. 2019; Newton et al. 2021). This persistence of *Salmonella* in broiler holdings can then serve as a reservoir for transmission and recurrent colonization of broilers and thus for contamination of meat at slaughter (Rose et al. 2000; Ethèves et al. 2021; Zeng et al. 2021).

In many countries, litter is not discharged from the poultry house before repopulation with the next flock occurs (de Toledo et al. 2020), thus persistence of pathogens is not unexpected during a fattening cycle. In Switzerland, however, litter is discharged from commercial poultry houses after each flock cycle per standard protocol, followed by cleaning and disinfection of the houses, downtime and spreading of new bedding before restocking (Neubauer et al. 2005; Mateus‐Vargas et al. 2022). *Salmonella* contamination of broiler houses occurs either by surviving bacteria in the empty house during downtime (Van Immerseel et al. 2009) or by entry of *Salmonella* persisting elsewhere in the integration or the environment of the farm. The causes are most often biosafety breaches by introduction of pathogens during thinning due to inadequate entry hygiene, or inadequate cleaning and disinfection at the end of the cycle. Recontamination with *Salmonella* from within the house or from the farm surroundings may reoccur after several months or even years (Rose et al. 2000).

Targeted measures to reduce human salmonellosis consist of *Salmonella* monitoring in food producing animals and products of animal origin. In Switzerland, the pillars of this are a mandatory national *Salmonella* control program, established in 1995, and the use of heat‐treated feed since 1996 (Hoop 1997). The mandatory control program follows a test‐and‐cull strategy, strictly without vaccination, and has proved successful to eliminate the target serovars (SE, STm, or mSTm) from egg and meat production, i.e. annually less than 2% prevalence in laying hens, and less than 1% prevalence in broilers and breeders (Anonymous 2024).

If environmental samples such as boot socks (fabric swabs) or feces test positive for *Salmonella* serovars SE, STm, or mSTm in laying hens, broilers or fattening turkeys, a stand‐still is imposed on the farm by Swiss veterinary authorities according to Swiss law (Anonymous 2021b). To corroborate a suspected salmonellosis case, culled animals from the affected flock are then examined in a veterinary laboratory accredited according to ISO/IEC 17025 (International Standard Organization 2005; Anonymous 2008). In case of detection of *Salmonella* in inner organs or musculature, the entire flock is culled. Veterinary authorities supervise the procedure including cleaning and disinfection measures, and a post‐disinfection control is performed by testing fabric swab samples from the poultry house before restocking (Anonymous 2021a). Alternatively, it would also be possible to slaughter a colonized flock, but the meat would have to undergo heat treatment, and this is not cost‐effective in Switzerland (Anonymous 2021b).

Contrary to the above‐described procedure, the flock is not culled, and veterinary authorities are further not involved if a non‐SE/non‐STm *Salmonella* serovar is detected in environmental samples. In case of broilers, cleaning and disinfection are done by the farmer or a cleaning company, followed by 3–5 days downtime. Because the legal focus is on SE and STm, farmers may not give the isolation of a non‐SE/non‐STm serious consideration, and the same serovar may be found in the boot sock sample during the next production cycle. Currently there is limited information about within‐flock persistence of non‐SE/STm *Salmonella* serovars in broiler farms. The methodology of whole‐genome sequencing (WGS) facilitates investigation into clonal *Salmonella* isolates, including persistence of distinct serovars in food production, genetic variation, source attribution and outbreak analysis (Yoshida et al. 2016; Horlbog et al. 2021; Kitchens et al. 2024).

The aim of this retrospective study was to assess the recurrence or persistence of *Salmonella* isolates at farm level. Four non‐SE/STm *Salmonella enterica* subsp. *enterica* serovars were assessed: *S*. Infantis, *S*. Livingston, *S*. Welikade and monophasic variant of *Salmonella enterica* subsp. e*nterica* 13,23:i:‐ (henceforth referred to as *S*. 13,23:i:‐) isolated from boot socks from 11 broilers houses from 10 commercial broiler farms over the course of months or years between 2013 and 2024. The study objectives were to prove whether the same clone persisted in the poultry house over the years, or whether similar but non‐clonal strains of the same serovar emerged over time. Further, sequence types (STs), antibiotic resistance genes and virulence factors were assessed through the obtained sequences.

## 2 Materials and Methods

### 2.1 Farms and Sampling

A total of 11 commercial broiler houses from 10 Swiss farms from two meat integrations A (farms 1 and 2) and B (farms 3–10) with recurrent *Salmonella* isolation of the same serovar from environmental (boot sock) samples were assessed (Table 1). Farm 2 of integration A comprised two broiler houses and a biogas plant present on the property. All holdings consisted of a broiler house with a hygiene entry and access to a winter garden. Each farm used conventional broilers (Ross 308 PM hybrid). Average placement capacity at the timepoint of boot sock sampling ranged from approximately 3960 to 25,000 (Table 1) in accordance with the legal maximum livestock populations allowed per farm in Switzerland, that is 27,000 broilers until the age of 28 days; 24,000 broilers from day 29 until day 35; 21,000 broilers from day 36 until day 42 or 18,000 broilers from day 43 onwards (Anonymous 2013). By law, maximum stocking density in Switzerland is 30 kg/m^2^ for conventional broilers (Anonymous 2008), which is less than the average 33–42 kg/m^2^ in the EU (Nielsen et al. 2023). The production cycle varies per farm according to the desired product demanded by the market: 28 days for poussins and up to five partial depopulations (thinning) until 36 days for other products (veterinarian of integration B, personal communication). Litter is always discharged between flocks, and the house is cleaned and disinfected by the poultry farmer according to the integration's standard protocol. In both investigated integrations, the cleaning and disinfection protocol consisted of several steps including dry and wet cleaning, disinfection and fogging.

**Table 1:** Identification of isolates, flock size and date of selected isolations of *Salmonella enterica* subsp. *enterica* serovars in 10 commercial broiler farms at two different time points and from several Swiss cantons.

Farm no.		1	2		3	4	5	6	7	8	9	10
Broiler integration		A			B							
*Salmonella* enterica serovars		*S*. Infantis	*S*. Livingston	*S*. Welikade	*S. enterica* subsp. *enterica* 13,23: i: ‐ (confirmed to be *S*. Idikan)							
Summary of the recurrent isolation on the farm	Number of isolates	5	4	7	10	2	5	2	2	1	1	1
	Time period	2021–2022	2019–2022	2019–2022	2013–2021	2017–2020	2022–2023	2022	2023	2023	2024	2024
Chosen early isolate for sequencing	Date (month/year)	05/2021	07/2019	09/2020	01/2014	11/2017	06/2022	09/2022	05/2023	06/2023	09/24	11/2024
	Flock size	5760	5550	3960	24,000	19,000	24,000	11,300	8900	18,500	16,700	25,000
	Strain ID NRGK^a^	21‐S2336	19‐S3012	20‐S3357	14‐S1170	17‐S3896	22‐S2852	22‐S3671	23‐S2504	23‐S2574	24‐S3648	24‐S4018
	Strain ID NENT^b^	N21‐1158	N17‐1695	N20‐1801	N17‐3031	N17‐3003	N22‐1429	N22‐2697	N23‐1196	N23‐1291	N24‐2544	N24‐3136
Chosen later isolate for sequencing	Date (month/year)	03/2022	03/2022	01/2022/08/2024	05/2020	05/2020	08/2023	11/2022	07/2023	na^c^	na	na
	Flock size	5490	7150	3960 and 7110	24,000	19,000	24,000	11,200	7800	na	na	na
	Strain ID NRGK	22‐S1614	22‐S1534	22‐S1126/21‐S3263^d^	30‐S2021	20‐S2201	23‐S3158	22‐S4018	23‐S2963	na	na	na
	Strain ID NENT	N22‐0509	N22‐0462	N22‐0149/N21‐2579^d^	N20‐0770	N20‐0864	N23‐1967	N22‐3227	N23‐1690	na	na	na
Swiss canton^e^		BE	BE	BE	TG	TG	AG	AG	AG	SH	SO	AG

^a^ Strain identification (ID) at National Reference Centre for Poultry and Rabbit Diseases (NRGK, University of Zurich, Switzerland).

^b^ Strain ID at National Reference Centre for Enteropathogenic Bacteria and Listeria (NENT, University of Zurich, Switzerland).

^c^ na: not applicable.

^d^ isolation from a second house on the same farm.

^e^ Swiss cantons: BE, Berne. TG, Thurgau. AG, Aargau. SH, Schaffhausen. SO, Solothurn.

Boot socks samples considered in this retrospective study had been taken by the poultry farmers according to standard sampling procedure (Anonymous 2021b) and had been processed at the National Reference Centre for Poultry and Rabbit Diseases (NRGK), University of Zurich, Switzerland, between 2013 and 2024. After detection of a non‐SE/STm *Salmonella* serovar in boot socks, none of the investigated broiler farms had performed a disinfection control by testing swab samples from the poultry house during downtime and before restocking, as non‐SE/STm *Salmonella* serovars are not regulated by veterinary authorities.

### 2.2 *Salmonella* Isolation and Serotyping

Isolation of *Salmonella* spp. was performed according to ISO 6579‐1:2017 (Anonymous 2017). Presumptive positive colonies were identified using matrix‐assisted laser desorption ionization‐time of flight mass spectrometry (MALDI‐TOF MS, Flex Control 3.4, MBT Compass 4.1.1, MBT Compass BDAL Library March 2023, Bruker Daltonics, 28359 Bremen, Germany), following the manufacturer's instructions. Serotyping was done according to the Kauffmann‐White‐LeMinor‐Scheme (Grimont and Weill 2007). This task is centralized in Switzerland according to a mandatory standard protocol, where serovars of isolates of animal origin are determined by the Swiss Reference Laboratory, the Center for Zoonoses, Animal Bacterial Diseases and Antimicrobial Resistance (ZOBA; University of Berne, Switzerland) (Anonymous 2021b). The majority of *Salmonella* isolated in the NRGK were stored at the National Reference Centre for Enteropathogenic Bacteria and Listeria (NENT; University of Zurich, Switzerland) at −80°C.

### 2.3 Whole‐Genome Sequencing Analysis

Recurrent *Salmonella* isolates (*n* = 20) from each broiler house from two time points were selected for sequencing (Table 1). The investigated isolates were selected to represent an early and late time point of data collection.

Stored *Salmonella* strains were streaked on Columbia agar with 7% sheep blood (Thermo Fisher Scientific, Waltham, MA, USA) and incubated at 37°C for 24 h.

DNA was extracted using the DNA blood and tissue kit (Qiagen, Hombrechtikon, Switzerland). Library preparation, sequencing and assembling were essentially performed as described previously (Stevens et al. 2024). In short, paired‐end DNA libraries were prepared using an Illumina DNA Prep, (M) Tagmentation Kit (Illumina, San Diego, CA, USA) and WGS was done on a MiniSeq sequencer producing 2 × 150 bp reads (Illumina, San Diego, CA, USA). Core genome MLST (cgMLST) was performed using the Ridom Seqsphere+ client version 10.0.6 with server version 10.0.1 and the EnteroBase *S. enterica* cgMLST v2 scheme.

The data of this project were deposited in BioProject PRJNA1230110. The Whole Genome Shotgun project was deposited at DDBJ/ENA/GenBank under the accessions JBLWPL000000000 to JBLWQF000000000. The versions described in this paper are versions BLWPL000000000 to JBLWQF000000000.

Published sequences of two O 13,23‐serovars were included in the analysis: strain FSIS12434301 of *Salmonella* Idikan (*Salmonella enterica* subsp. *enterica* 1,13,23:i:1,5) and strain FSIS12534671 (SA613) of *Salmonella* Kedougou (*Salmonella enterica* subsp. *enterica* 1,13,23:i:l,w).

Persistence of the same strain of a serovar within a poultry house over time was indicated by isolates of the same serovar clustering into the same clonal group, with a cut‐off of < 8 cg alleles.

Serotyping was confirmed by the *Salmonella In Silico* Typing Resource (SISTR) (Yoshida et al. 2016).

Antimicrobial resistance genes were identified using the Resistance Gene Identifier (RGI) 6.0.5 and database version 4.0.1(Alcock et al. 2020), downloaded in April 2025. The cut‐off was > 80% identity on amino acid level over > 90% of the length.

Virulence factors were identified using a bidirectional‐best‐hit approach with diamond (Buchfink et al. 2021), using the core protein data set A from the virulence factor database (Liu et al. 2022) and the predicted proteomes of the strains as input. The cut‐off was 70% identity. All databases for bioinformatical analyses were downloaded in July 2025.

## 3 Results

A total of 40 *Salmonella* isolates were collected during routine diagnostic testing of 11 poultry houses from 10 broiler farms between 2013 and 2024 (Table 1). *Salmonella enterica* subsp. *enterica* serotypes were identified using the White‐Kauffmann‐Le Minor scheme, and were later confirmed by WGS and serotyping by SISTR. Twenty‐four isolates belonged to *S*. 13,23:i:‐, 7 to *S*. Welikade, 5 to *S*. Livingston and 4 to *S*. Infantis.

**Figure 1:**
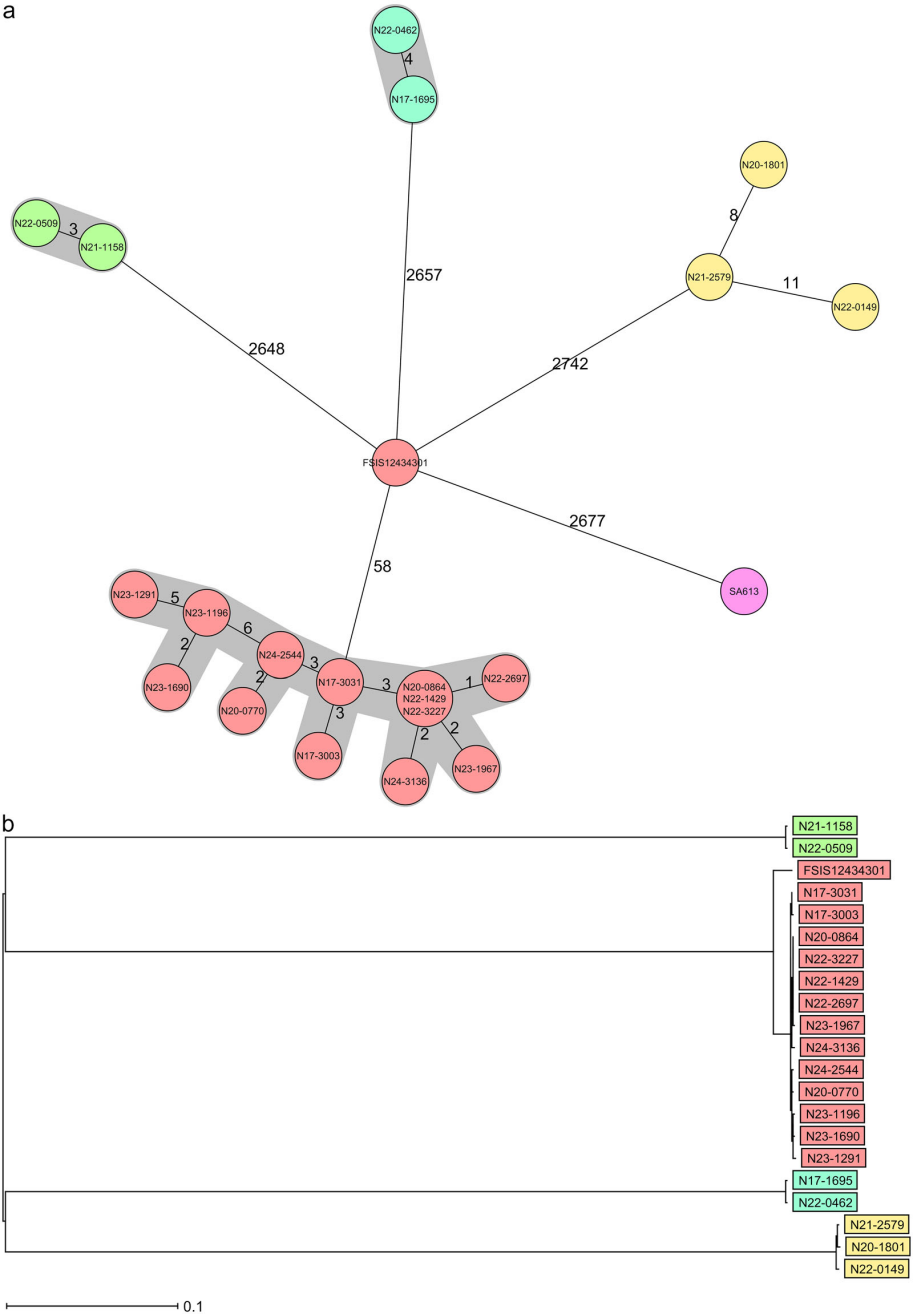
(a) cgMLST based minimum‐spanning tree of *Salmonella* isolates (*n* = 20 samples) from 10 farms as well as reference strains of *Salmonella enterica* subsp. *enterica* serovar Idikan (*S*. Idikan; strain FSIS12434301) and *Salmonella enterica* subsp. *enterica* serovar Kedougou (*S*. Kedougou; strain FSIS12534671 (SA613)) using Ridom SeqSphere + . Each circle represents an allelic profile based on target core genome MLST. The numbers between connected circles represent the allelic differences between two strains with a clonal cut‐off < 8. Each circle contains the strain ID. The label colors denote the isolate source. Sequence type (ST): ST32 (green); ST457 (turquoise); ST1891 (red); ST3300 (yellow) and ST469 (magenta). (b) Maximum‐likelihood phylogenetic tree based on SNPs of *Salmonella* isolates of this study and including *Salmonella* enterica subsp. enterica serovar Idikan (*S*. Idikan; strain FSIS12434301) as a reference strain.

Whole‐genome sequencing analysis revealed that the isolates of each serovar belonged to the same STs and to specific clonal clusters with < 7 cg allele differences: *S*. Infantis ST32, *S*. Livingston ST457 and *S*. 13,23:i:‐ ST1891 (Figure 1a). All *S*. 13,23:i:‐ monophasic variant isolates derived from farms belonging to the same broiler integration (B), and were clonal within and among all investigated farms (< 7 cg alleles) over the course of 11 years.

Most isolates originated from farm no. 3 with seven isolates persisting in the broiler house during the years 2014 and 2015, followed by one isolate only each year in 2018 and 2020.

In addition, a cgMLST comparison of *S*. 13,23:i:‐ revealed a distance of 58 alleles to *S*. Idikan strain FSIS12434301 and of 2677 alleles to *S*. Kedougou FSIS12534671 (SA613), thus attributing the monophasic *S*. 13,23:i:‐ variant to *Salmonella enterica* serovar Idikan.

Isolates of *S*. Welikade (ST3300) from broiler integration (A) detected in 2020, 2021, and 2022 had 8 and 11 cg allele differences among isolates (Figure 1a), indicating very similar but non‐clonal strains in the poultry houses (*n* = 2) of the same broiler farm.

In accordance with the data above, all clonal strains tightly clustered in an SNP‐based tree analysis (Figure 1b).

The presence of complete genomes allowed for analyses of antimicrobial resistance genes. Antimicrobial resistance genes for efflux pumps (*mdsA/mdsB*) were detected in all investigated isolates, while fosfomycin (*fosA7.3*) was identified only in isolates from integration B (Table 2).

**Table 2:** Antimicrobial resistance genes identified in *Salmonella enterica* subsp. *enterica* isolates in 10 Swiss commercial broiler farms of integrations A and B.

*Salmonella enterica* serovars	Broiler integration	Farm no.	Strain ID^a^	ST^b^	cgMLST^c^	Efflux	Fosfomycin
*S*. Infantis	A	1	N21‐1158	32	29,753	mdsA/mdsB	
			N22‐0509	32	29,753	mdsA/mdsB	
*S*. Livingston		2	N17‐1695	457	29,750	mdsA/mdsB	
			N22‐0462	457	29,750	mdsA/mdsB	
*S*. Welikade			N20‐1801	3300	10,401	mdsA/mdsB	
			N22‐0149	3300	29,754	mdsA/mdsB	
			N21‐2579	3300	10,401	mdsB	
*S. enterica* subsp. *enterica* 13,23: i: ‐ (*S*. Idikan)	B	3	N17‐3031	1891	27,169	mdsA/mdsB	fosA7.3
			N20‐0770	1891	29,752	mdsA/mdsB	fosA7.3
		4	N17‐3003	1891	27,169	mdsA/mdsB	fosA7.3
			N20‐0864	1891	27,169	mdsA/mdsB	fosA7.3
		5	N22‐1429	1891	29,755	mdsA/mdsB	fosA7.3
			N23‐1967	1891	29,755	mdsA/mdsB	fosA7.3
		6	N22‐2697	1891	29,755	mdsA/mdsB	fosA7.3
			N22‐3227	1891	29755	mdsA/mdsB	fosA7.3
		7	N23‐1196	1891	29,752	mdsA/mdsB	fosA7.3
			N23‐1690	1891	29,752	mdsA/mdsB	fosA7.3
		8	N23‐1291	1891	29,756	mdsA/mdsB	fosA7.3
		9	N24‐2544	1891	27,169	mdsA/mdsB	fosA7.3
		10	N24‐3136	1891	29,755	mdsA/mdsB	fosA7.3

^a^ Strain identification (ID) at National Reference Centre for Enteropathogenic Bacteria and Listeria (NENT, University of Zurich, Switzerland).

^b^ ST: Sequence type.

^c^ cgMLST: core genome multilocus sequence typing.

Virulence factors were also identified. The majority of the genes (217/231) were present in all 20 isolates and included genes involved in adhesion, invasion, and intracellular survival of *Salmonella* during infection (Table S1). For example, all isolates contained curli fimbriae adhesion genes, *fimF*, *sipA*, *sopA, sseB, mgtA/B*, and *pipB*, among others (Table S1).

To check whether the study strains are related to other strains, the genomes were compared to genomes in public databases. A cgMLST‐based comparison of the study isolates to all isolates available at cgMLST. org (*n* = 29816), revealed no clonal relation to any existing strains (data not shown).

## 4 Discussion

Since introducing national control programs in Switzerland in 1995 and EU countries in 2008, the prevalence of STm/SE serovars has been very effectively reduced, while the prevalence of non‐SE/STm serovars has remained relatively constant (Arnold et al. 2023; Anonymous 2024). According to Arnold et al. 2023, the prevalence of all non‐SE/STm serovars taken together was estimated at 1.6% (95% confidence interval (CI) 1.0%–5.9%) as compared to SE/STm serovars 0.20% (95% CI 0.11%–0.87%) in 2018. More specifically, approximately 95% of *S*. Infantis isolates originate from the broiler sector in the EU, and in parallel, this serovar ranks as the fourth most prevalent serovar associated with human salmonellosis (Alvarez et al. 2023; Montoro‐Dasi et al. 2023).

Currently, the gold standard for *Salmonella* serovar identification is serotyping according to the White‐Kauffman‐LeMinor scheme, which is based on immunological reactions to somatic (O) and flagellar (H) antigens (Guibourdenche et al. 2010). However, this method lacks the resolution power to disclose whether isolates from the same serovar can be attributed to the same strain, which it is critical for epidemiological surveillance and source investigation (Yachison et al. 2017; Diep et al. 2019). WGS was thus performed to characterize the diversity and clonal relationships of the *Salmonella* study isolates from different poultry houses over time, with a cut‐off < 8 cg alleles based on sub‐typing methods. WGS represents the method of choice with a fast turnaround time and acceptable costs to identify subtle differences between highly clonal strains (Nadon et al. 2017; Antony et al. 2020; Horlbog et al. 2021).

For integration A, survival and persistence of a farm‐specific clone was shown in the current study in cases of *S*. Infantis and *S*. Livingston. For *S*. Welikade, very closely related, but non‐clonal strains could be demonstrated on a poultry farm with a biogas plant on the premises, representing either an evolving persistent strain or reintroduction of very similar strains into the broiler houses. Dispersal within integration A was not evident for any of these three serovars. Samples from the hatchery, broiler breeder and other broiler facilities examined in the NRGK were negative at any given time point, and further internally obtained data were also confirmed as negative by the integration veterinarian (data not shown). All study strains of these three serovars were shown not to belong to well‐known outbreak strain clusters present in public databases, in fact, they were not clonally related to any existing strains therein.

In case of integration B, spread of *S*. 13,23:i:‐ within the broiler integration was disclosed by WGS, which also unveiled a close relationship of *S*. Idikan to the *S*. 13,23:i:‐ study isolates, with only 58 alleles differences, presumably placing the monophasic variant *S*. 13,23:i:‐ into serovar Idikan. Similar to the finding in this study of a dispersal of *S*. 13,23:i:‐ within a broiler integration, a monophasic variant of *S*. Idikan was recently isolated from several feed mills and hatcheries and correlated with an infection in both broiler breeding and broiler flocks in the United Kingdom from 2016 to 2019, even though this serovar is not be expected to be vertically transmitted (Koutsoumanis et al. 2019; Oastler et al. 2022). *S*. Idikan has also been isolated from laying hens in Chad (Tabo et al. 2013) and from livestock and poultry on farms, meat at abattoirs, raw materials at feed mills, animal feed, and environmental sources in South Africa (Magwedere et al. 2015). *S*. Idikan was proved particularly difficult to eliminate if present in poultry production (Oastler et al. 2022). This, in turn, may explain the frequent circulation of *S*. 13:23:i:‐ within integration B. Additionally, the high clonal relationship among isolates of integration B indicates that the clone was either transmitted between farms by means of transport vehicles or by introduction and spreading within the broiler integration company, which strongly suggests a cross‐contamination throughout the same integrated company over the course of 11 years. The study strains did not show a clonal relationship to any strains in the databases, therefore the origin of this persistent *S*. Idikan strain remains unknown.

Recently, survival of an identical strain of *S*. Enteritidis in slaughterhouses was shown to contribute to cross‐contamination throughout the broiler supply chain and slaughter stages, which was mainly explained by insufficient cleaning and disinfection procedures (Zeng et al. 2021; Marin et al. 2022). Efficient cleaning and disinfection practices of broiler houses are essential for biosecurity and aim to avoid risk of persistence (Payne et al. 2005; Burbarelli et al. 2015). The current investigations show that a single clone of a non‐SE/STm *Salmonella* serovar is able to persist on a farm or can be dispersed to the farms repeatedly within an integration for several years. In case of farm 3, re‐isolation occurred over the course of 9 years.

The unremarkable antimicrobial resistance pattern of the study strains remained stable over the timeline from 2013 until 2024, indicating that they likely represent local strains without evolutionary pressure to become increasingly resistant to antimicrobials, as flocks are not treated for *Salmonella*. Moreover, Switzerland promotes the prudent use of antibiotics in broiler flocks, with only one in 10 flocks receiving an antibiotic treatment once during rearing, and merely 0.8% of the total antibiotics used in Swiss veterinary medicine used to treat poultry in 2020 (Anonymous 2021b). In all 4 serovars only efflux pumps (*mdsA*/*mdsB* genes) were found. In contrast, a high prevalence of diverse efflux pump encoding genes among antibiotic resistant *S*. Infantis isolates have been reported in Brazil (Vilela et al. 2022).

The *fosA7.3* gene conveying resistance to fosfomycin was found exclusively among *S*. Idikan. Taken together, this is a very different antibiotic resistance situation compared to that in *Salmonella* from broilers from e.g. Spain (Cortés et al. 2022). In particular, the herein described *S*. Infantis ST32 strains of do not compare to the emerging multidrug resistance *S*. Infantis ST32 strains harboring the “plasmid of emerging *S*. Infantis” (pESI). This plasmid contains genes coding for resistance to tetracycline, trimethoprim, streptomycin, sulfamethoxazole, and tetracycline, and is also present in imported Swiss poultry meat at retail level (Hindermann et al. 2017; Barmettler et al. 2025). Nor are they similar to Dutch *S*. Infantis ST32 strains (Mughini‐Gras et al. 2021). The origin of *S*. Idikan with a resistance to fosfomycin, an antibiotic not used in Swiss broiler flock, could not be elucidated, because testing within integration B was not possible. Recently, a high prevalence of fosfomycin resistance genes was found among multiple *Salmonella* serovars, highlighting a potentially serious public health concern (Monte et al. 2023).

The high number of virulence genes and the overall presence of important virulence factors involved in infection strongly suggest that all investigated isolates have a potential to cause salmonellosis in humans (Li et al. 2025). Similarly, evidence of dissemination of virulent and multidrug‐resistant *S*. Infantis were found in poultry meat (Lapierre et al. 2020).

The persistence of non‐SE/STm *Salmonella* serovars in broiler houses demonstrated in the current study should give rise to further control measures related to (i) additional criteria such as efficient biosecurity measures of non‐SE/STm serovars to avoid on‐farm and farm‐to‐farm contamination, (ii) efficient cleaning and disinfection protocols for broiler facilities after each flock cycle, with a special focus on critical locations, such as drinking nipples, floor cracks, ventilation systems and drains (Luyckx et al. 2015; Martelli et al. 2017). For instance, it has been verified previously that cracked floors are difficult to clean and have higher *Salmonella* prevalence than intact floors (Mueller‐Doblies et al. 2010). Conducting fabric swab sample testing of the cleaned poultry house before restocking would be advisable.

## 5 Conclusions

Identical clones of non‐SE/STm *Salmonella* serovars, repeatedly isolated from the same farms over months and years, but not clonally related to any genome sequences of *Salmonella* in public databases, were identified by WGS and SNP‐based tree analysis, suggesting their persistence in the environment of broiler farms. Thus, an effective cleaning and disinfection practice before restocking is essential to ensure that the next flock is not contaminated with non‐SE/STm *Salmonella* serovars. Results from this study may help to shed some light on the dynamics of persistence and reintroduction of *Salmonella* serovars other than SE and STm in broiler houses and are therefore important for on‐farm biosafety measures and public health.

## Author Contributions

**Maher Alsaaod:** conceptualization, writing – original draft, writing – review and editing. **Marc J. A. Stevens:** methodology, formal analysis, data curation. **Nicole Cernela:** investigation. **Jule Anna Horlbog:** investigation. **Roger Stephan:** writing – reviewing and editing, resources. **Sarah Albini:** conceptualization, writing – original draft, writing – review and editing, supervision.

## Ethics Statement

The authors have nothing to report.

## Conflicts of Interest

The authors declare no conflicts of interest.

## Supporting information


**Table S1:** Distribution of virulence genes detected in 20 *Salmonella enterica* subsp. *enterica* isolates from Swiss broiler farms.

## Data Availability

The Whole Genome Shotgun project has been deposited at DDBJ/ENA/GenBank under the accessions JBLWPL000000000 to JBLWQF000000000. The versions described in this paper are versions BLWPL000000000 to JBLWQF000000000. All data relevant to the study are included in the article or are enclosed in the supporting information.
